# AI-Integrated QSAR Modeling for Enhanced Drug Discovery: From Classical Approaches to Deep Learning and Structural Insight

**DOI:** 10.3390/ijms26199384

**Published:** 2025-09-25

**Authors:** Mahesh Koirala, Lindy Yan, Zoser Mohamed, Mario DiPaola

**Affiliations:** Therabene Inc., Norwood, MA 02062, USA; lyan@therabene.com (L.Y.); zmohamed@therabene.com (Z.M.)

**Keywords:** QSAR modeling, artificial intelligence, machine learning, deep learning, molecular docking, molecular dynamics, PROTACs, ADMET prediction, cheminformatics, drug discovery, graph neural networks

## Abstract

Integrating artificial intelligence (AI) with the Quantitative Structure-Activity Relationship (QSAR) has transformed modern drug discovery by empowering faster, more accurate, and scalable identification of therapeutic compounds. This review outlines the evolution from classical QSAR methods, such as multiple linear regression and partial least squares, to advanced machine learning and deep learning approaches, including graph neural networks and SMILES-based transformers. Molecular docking and molecular dynamics simulations are presented as cooperative tools that boost the mechanistic consideration and structural insight into the ligand-target interactions. Discussions on using PROTACs and targeted protein degradation, ADMET prediction, and public databases and cloud-based platforms to democratize access to computational modeling are well presented with priority. Challenges related to authentication, interpretability, regulatory standards, and ethical concerns are examined, along with emerging patterns in AI-driven drug development. This review is a guideline for using computational models and databases in explainable, data-rich and profound drug discovery pipelines.

## 1. Introduction

**Figure 1:**
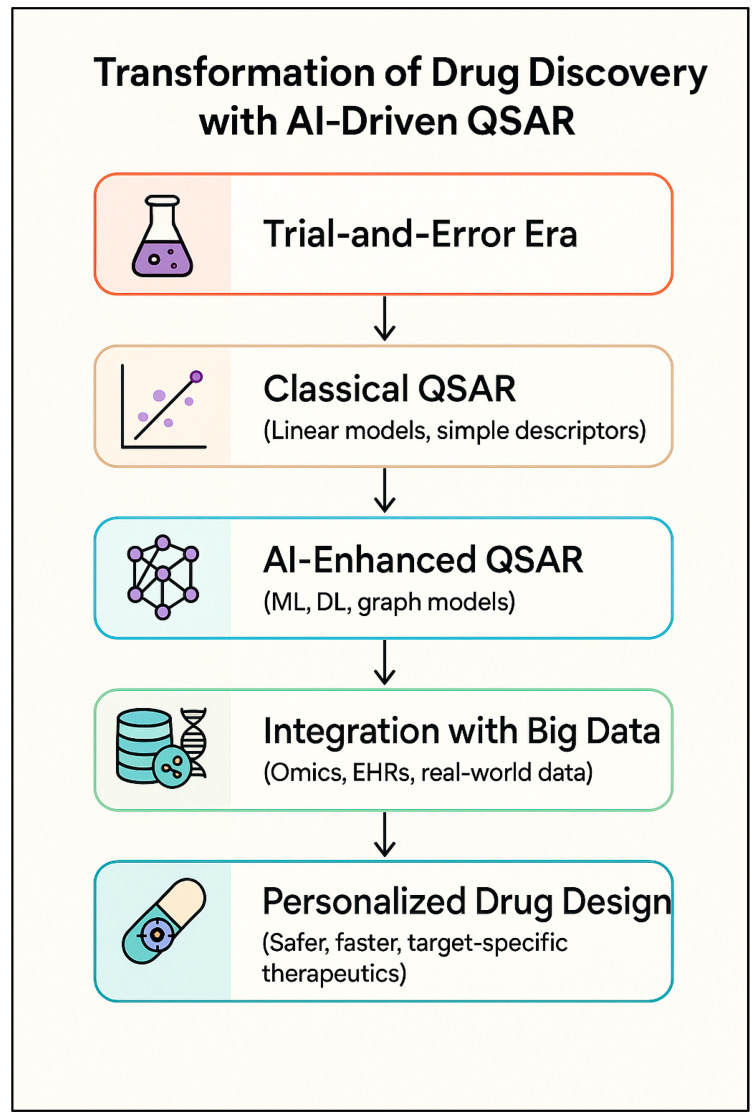
Overview of the shift from the trial-and-error methods to the personalized drug design, ranging from the classical approach to the modern AI techniques.

Drug discovery is undergoing a significant revolution, driven by integrating artificial intelligence (AI) into Quantitative Structure-Activity Relationship (QSAR) modeling [1,2,3,4]. Until recently, drug discovery was conducted primarily by trial-and-error, an approach that was time-consuming and burdened with high costs; currently, drug development is increasingly shaped by data-driven computational methodologies that should lead to speedier drug discovery and, ultimately, safer drugs as shown in Figure 1. Advances in QSAR have evolved from basic linear models to sophisticated machine learning (ML) and deep learning (DL) frameworks that integrate complex nonlinear patterns across large chemical spaces [1,5,6,7].

QSAR predictive power, when properly combined with AI, can facilitate virtual screening of extensive chemical databases, containing billions of compounds, de novo drug design and lead optimization for specific targets. Algorithms incorporating neural networks, generative models and reinforcement learning are reshaping how compounds are selected, modified, and evaluated for defined targets. Furthermore, integrating omics data, real-world evidence from medicine, and multi-parametric optimization pushes the frontier of personalized medicine and targeted therapeutics [8,9].

This article aims to explore the current trends in AI-augmented QSAR methodologies, highlighting key breakthroughs, and outlining emerging trends that could reshape the future of discovery and development of novel pharmaceutical products, especially with respect to small molecule compounds, such as inhibitors, degraders (PROTACS) and other modalities [9,10]. With the prospects of improving hit-to-lead timelines to designing safer and more effective drugs, the synergy between QSAR and AI is becoming the new foundation in modern drug discovery and development [11]. Recent applications highlight the role of AI-enhanced QSAR in real-world discovery. Talukder et al. integrated docking, QSAR, and simulations to investigate EGFR-targeting phytochemicals in non-small cell lung cancer [12]; Kaur et al. developed BBB-permeable BACE-1 inhibitors for Alzheimer’s disease using 2D-QSAR, docking, ADMET, and MD [13]; Souza et al. combined ML approaches and QSAR to analyze *SARS-CoV-2* Mpro inhibitors [14]; and Maliyakkal et al. applied QSAR-driven virtual screening to identify potential therapeutics against *Trypanosoma cruzi* [15]. These case studies illustrate how QSAR continues to advance modern inhibitor discovery.

Computational approaches significantly accelerate the preclinical stage of drug discovery by reducing costs, minimizing attrition, and expediting the identification of viable candidates. Ou-Yang et al. described how computational pipelines streamline hit-to-lead discovery, reducing reliance on expensive high-throughput screening [16]. More recently, Ouma et al. reviewed modern computational approaches and emphasized their role in decreasing attrition rates and supporting ADMET profiling in early discovery [17]. In parallel, Lavecchia highlighted how virtual screening and QSAR methodologies have become central tools in prioritizing compounds before synthesis, directly cutting down experimental costs [18]. Similarly, Paul et al. demonstrated that computational filtering and optimization strategies reduce the time required to progress leads into the preclinical pipeline, showing tangible benefits in pharmaceutical R&D [19]. These studies collectively demonstrate that computational drug discovery is not only complementary to laboratory methods but also indispensable for modern, cost-effective preclinical development.

## 2. Foundations of QSAR and Molecular Descriptors

QSAR modeling is dependent on molecular descriptors, which are numerical values that encode various chemical, structural, or physicochemical properties of compounds. The descriptors are generally classified according to dimensions as 1D, 2D and 3D that correspond to the compound’s properties like molecular weight, topological indices and molecular shape or electrostatic potential map, respectively. To increase the model efficiency and reduce overfitting, dimensionality reduction techniques such as principal component analysis (PCA) and recursive feature elimination (RFE) are being highly used [7,20]. The appropriate selection and interpretation of these descriptors are necessary for making predictive, robust QSAR models. There are even more sophisticated methods, including LASSO (Least Absolute Shrinkage and Selection Operator), and mutual information ranking. They are being used frequently to eliminate irrelevant or redundant variables and to identify the most significant features [21,22,23]. These methods not only improve model performance but also enhance interpretability, which is essential for governing approval and hypothesis generation in medicinal chemistry. In addition to 1D, 2D, and 3D descriptors, 4D descriptors have also been developed. These account for conformational flexibility by considering ensembles of molecular structures rather than a single static conformation. Ensemble-based descriptors provide more realistic representations of molecules under physiological conditions and have been applied in ligand-based pharmacophore modeling and QSAR refinement [24,25].

Furthermore, Quantum chemical descriptors like the HOMO-LUMO gap, dipole moment, molecular orbital energies, and electrostatic potential surfaces have also found extensive application in QSAR modeling, particularly for drug-like molecules where electronic properties influence bioactivity [1,9]. The use of 3D descriptors such as molecular surface area, volume, and conformer-based properties has expanded with the availability of tools like DRAGON, PaDEL, and RDKit [26,27,28]. Likewise, the latest integration of deep learning techniques has led to the development of learned molecular representations, or “deep descriptors,” which are derived from molecular graphs or SMILES strings without manual descriptor engineering [29]. These latent embeddings created by graph neural networks (GNNs) or autoencoders are capable of capturing more abstract and hierarchical molecular features, which opens new possibilities for constructing data-driven, flexible QSAR pipelines applicable across diverse chemical spaces [30,31].

## 3. Classical QSAR: Statistical Modeling Techniques

In classical QSAR, the molecular descriptors are correlated with biological activity by statistical regression method. Other traditional but effective approaches used extensively in drug discovery and environmental toxicology includes Multiple Linear Regression (MLR), Partial Least Squares (PLS), and Principal Component Regression (PCR). These approaches are esteemed for their simplicity, speed, and ease of explanation, specifically in governing settings. They are generally practical when a reasonably small number of variables show linear relationships with the biological response. For model validation, internal metrics such as R^2^ (coefficient of determination) and Q^2^ (cross-validated R^2^), as well as external datasets that test the model’s generalizability on unseen compounds, are used. These models while being mostly dependent on assumptions of linearity, normal distribution, and independence among variables, which might not be true in large and chemically varied datasets [7,32,33].

Several efforts have been made over the years to boost the capabilities of classical QSAR models by incorporating robust feature selection and data preprocessing tactics. Several methods, like stepwise regression, bootstrapping, and residual analysis, have been initiated to expand stability and diminish overfitting [34,35]. Nevertheless, these models frequently falter while dealing with extremely nonlinear relationship or noisy data that cannot be modeled with simple parametric equations. Subsequently, hybrid approaches that combine the classical statistical tools with machine learning methods like PLS, combined with decision trees or ensemble averaging, have been developed to narrow this gap when gaining interpretability [11,36]. Despite the upwelling in popularity of complex models like random forests and deep learning, classical QSAR remains obligatory for preliminary screening, mechanism clarification, and when explainability is a greater priority, as in regulatory toxicology and REACH compliance [37]. Additional software packages like QSARINS and Build QSAR endure to support classical model development with enriched validation roadmaps and visualization tools [7,38].

Classical QSAR remains highly relevant in modern drug discovery when combined with rigorous validation. Olenginski et al. applied QSAR to RNA-binding small molecules, uncovering structural determinants of RNA–ligand recognition [39]. Talukder et al. integrated classical QSAR, docking, and simulations to prioritize EGFR-targeting phytochemicals for non-small cell lung cancer [12]. Kaur et al. designed BBB-permeable BACE-1 inhibitors using 2D-QSAR for Alzheimer’s disease, highlighting how traditional QSAR supports lead design [13]. Finally, Cherkasov et al. provided a landmark review demonstrating how classical QSAR principles underpin contemporary drug discovery pipelines [1]. These cases illustrate the enduring value of classical QSAR.

## 4. Machine Learning Rise in QSAR

Machine learning has significantly increased the predictive influence and flexibility of QSAR models, mainly in managing complex, high-dimensional chemical datasets. Algorithms like Support Vector Machines (SVM), Random Forests (RF), and k-Nearest Neighbors (kNN) are the most standard tools in cheminformatics and are widely used for tasks ranging from virtual screening to toxicity projection [40,41,42]. Unlike classical linear models, these algorithms can successfully capture nonlinear relationship between molecular descriptors and biological activity without earlier assumptions about data distribution. Generally, Random Forests are preferred for their robustness, built-in feature selection, and ability to handle noisy data, while SVMs are more efficient in conditions with regulated samples and high descriptor-to-sample ratios [43,44,45]. This is because of the several reasons. RF can manage irrelevant or redundant descriptors because its random feature selection at each split reduces the risk of overfitting to noisy variables, it tolerates collinearity among descriptors, since each tree considers only a subset of variables. and it demonstrates resilience to outliers in bioactivity data, as the ensemble averaging across many trees dampens the effect of anomalous points [46,47]. Grid search and Bayesian optimization are other hyperparameter optimization strategies that are regularly applied to fine-tune these models for top predictive performance.

Modern developments have also focused on boosting interpretability and decreasing the “black-box” nature of machine learning in QSAR. Feature importance ranking methods like permutation importance, SHAP (SHapley Additive exPlanations), and LIME (Local Interpretable Model-agnostic Explanations) now allow researchers to comprehend which descriptors influence model predictions the most [48]. Ensemble learning methods such as stacking, bagging, and boosting have further improved model stability and precision across diverse chemical spaces. These novelties, combined with increasing access to curated datasets and open-source platforms which including scikit-learn, KNIME [49], and AutoQSAR, have democratized machine learning-based QSAR modeling [50]. Despite these encroachments, careful consideration of justification, applicability domain, and dataset bias remains indispensable to evade overfitting and confirm regulatory acceptance, principally in safety-critical domains like pharmacovigilance and environmental hazard prediction [51]. Machine learning has rapidly expanded QSAR capabilities. Singh et al. developed ML-guided QSAR for imidazole scaffolds, achieving strong predictive accuracy even in noisy datasets [42]. Souza et al. combined ML and QSAR to reveal antagonistic trends in *SARS-CoV-2* Mpro inhibitors, illustrating ML’s role in antiviral discovery [14]. Maliyakkal et al. performed QSAR-driven virtual screening for *Trypanosoma cruzi* therapeutics, showing the utility of ML-enhanced QSAR in neglected tropical diseases [15]. Zhang et al. demonstrated the power of ML-based QSAR for kinase inhibitor optimization, highlighting broader applicability across target classes [52]. Together, these studies show how ML augments QSAR with robustness and generalizability

## 5. Deep Learning and Neural Models in Drug Discovery

Deep learning has meaningfully renovated QSAR modeling by empowering automated feature extraction and representation learning right from raw molecular structures. Contrasting traditional models that depend on pre-calculated descriptors, deep learning architectures can generate molecular graphs, SMILES strings, and even 3D conformations to come up with abstract, high-level features relevant to biological activity prediction [9,53,54]. Convolutional Neural Networks (CNNs) have been adapted to operate on molecular grids and images, while Recurrent Neural Networks (RNNs), principally Long Short-Term Memory (LSTM) networks, are compatible with sequential representations like SMILES strings [55,56]. Another emerging field is Graph Neural Networks (GNNs), which have been particularly powerful tools in cheminformatics, as they natively model atoms as nodes and bonds as edges, perfectly reflecting the topology of molecular structures. Other tools like DeepChem [57], Chemprop [58], and DGL-LifeSci have also made these models more straightforward to non-experts [30].

Likewise, another most groundbreaking development in this area is the rise of chemical language models, such as SMILES-based transformers and autoencoders. The models, such as ChemBERTa [59] and MolBERT [60], are trained on millions of chemical structures and can be adjusted for explicit tasks such as activity projection, retrosynthesis, or de novo molecular generation. To propose novel compounds with enhanced activity and ADMET properties, Generative models that include Variational Autoencoders (VAEs) and Generative Adversarial Networks (GANs) are now commonly employed. One example includes REINVENT, which pools reinforcement learning with RNN-based generators to enhance molecules toward a predefined goal iteratively [61]. Deep Learning models in QSAR also encounter issues in interpretability, data disparity, and transferability within distinctive chemical domains. To solve these challenges, several techniques like attention mechanisms, multi-task learning, and data reinforcement approaches are being incorporated into pipelines to refine generalizability and cut overfitting [62,63,64]. Deep learning enables abstraction of complex features beyond the reach of classical QSAR. Li et al. developed DeepPROTACs, a deep learning framework for PROTAC design [65]. Goh et al. pioneered deep learning models for compound–protein interaction prediction, outperforming conventional QSAR [66]. Kim et al. applied SMILES-based deep networks for bioactivity prediction, achieving superior accuracy in chemical space coverage [67]. Altae-Tran et al. demonstrated one-shot learning for drug discovery, highlighting DL’s ability to learn from extremely limited data [68]. These advances underscore how DL reshapes QSAR by capturing nonlinear, high-dimensional representations.

## 6. Molecular Docking and Dynamics

Molecular docking is a powerful tool in drug discovery that simulates the interaction between ligands and target proteins, yielding the binding poses and estimating binding affinities through their scoring functions. It offers a critical first look at how a small molecule might bind to a receptor and is essential for virtual screening, lead identification, and understanding structure-activity interactions. The most commonly used docking platforms include AutoDock Vina (v1.1.2) [69], Glide (2004 release) [70], GOLD (v5.2) [71], and LeDock (2015 release) [72]. Each docking method differs in sampling techniques and scoring algorithms [73], with its binding affinities and pose geometries that can be further incorporated into QSAR models as molecular descriptors for improving predictive performance and interpretability. Besides its popularity and accessibility, it has some discrepancies as it treats receptors as rigid, and scoring functions usually might not completely capture entropic and solvent effects. While rigid docking assumes a fixed receptor structure, most modern approaches employ semi-flexible docking (ligand flexible, receptor side chains partially flexible) or flexible docking (both ligand and receptor sampled). These methods better capture induced-fit effects but also present limitations. First, flexible docking is computationally intensive, often requiring significant resources for conformational sampling. Second, it provides only a limited representation of receptor flexibility, as most algorithms sample a subset of side chains or backbone motions rather than the full conformational landscape. Third, flexible docking can increase the risk of false positives, since greater conformational freedom may allow unrealistic ligand poses without sufficient energetic penalties [73,74]. To address these limitations, flexible docking is frequently combined with molecular dynamics simulations, which provide a more rigorous account of receptor flexibility.

Molecular Dynamics (MD) simulations, which offer atomistic insights into the temporal behavior of protein-ligand complexes in a fully solvated environment, are used to overcome the limitations of docking tools. MD simulations help to determine the structural and conformational dynamics of protein-ligand complexes, which include pose stability, protein flexibility, and significant non-covalent interactions such as hydrogen bonds, salt bridges, van der Waals, and electrostatic interactions, which might be unnoticed in static docking studies. Software packages like GROMACS (v5.x), AMBER (v12/14), NAMD (v2.6) and CHARMM (CHARMM36) are commonly used to simulate biological systems on timescales ranging from nanoseconds to microseconds [75,76,77,78,79]. There are different commercial packages available for easy and fast MD simulations. The simulation results are combined with MM/PBSA or MM/GBSA methods to estimate binding free energies more precisely. The docking and MD are then combined with QSAR or ML/DL frameworks, resulting in the hybrid models that achieve higher performance in predicting binding affinity and action mechanisms [80]. It is widely known that integrating MD-derived features like root-mean-square deviation (RMSD), hydrogen bond analysis, and conformational clustering [81,82,83] can considerably advance both regression and classification outcomes in modern drug discovery pipelines [84,85]. Docking and molecular dynamics (MD) simulations complement QSAR by providing mechanistic insight into ligand–target interactions. Koirala and Fagerquist combined MD with QSAR to probe Colicin–Immunity protein complexes [81]. Lu et al. applied long-timescale MD to GPCRs, revealing conformational states essential for ligand binding [86]. Hollingsworth and Dror reviewed how MD simulations guide drug discovery across multiple protein classes, demonstrating predictive power at atomic resolution [75]. Pagadala et al. provided a comprehensive review of docking applications, emphasizing the strengths and limitations of docking tools in lead optimization [73]. These cases illustrate how docking and MD extend QSAR into structure-based discovery.

## 7. PROTACs and Targeted Protein Degradation

Proteolysis-targeting chimeras (PROTACs) or degraders represent a highly promising therapeutic strategy that goes beyond inhibition by eliminating disease-causing proteins by harnessing the ubiquitin-proteasome system. These bifunctional molecular constructs bind to a target protein and an E3 ubiquitin ligase, concurrently, forming a ternary complex that leads to ubiquitination and subsequent proteolysis of the target protein. This mechanism unlocks potential access to previously “undruggable” proteins and opens new avenues for treating cancer, neurodegenerative diseases, and immune disorder. PROTACs’ development presents several challenges due to the size, flexibility, and reliance on complex protein–protein interactions of these molecular constructs. Indeed, existing drug discovery tools are not very effective at accurately predicting degraders’ pharmacokinetics, efficacy, or degradation behavior [87,88,89].

Most degraders are over 800 Da in molecular weight, display high flexibility, and contain three distinct substructures: a moiety specific to the target protein, a linker, and an E3 ligase binder. This modular structure creates complex, nonlinear relationship between chemical structure and biological activity. Existing QSAR models rely on relatively static structure–activity correlations and thus are not well suited to capture multi-factorial interactions. Descriptors, including LogP, molecular size, and polar surface area (PSA), are insufficient for prediction of critical degrader determinants, such as ternary complex stability, target-E3 ligase cooperativity, linker geometry and flexibility and cell permeability and metabolic stability [90,91,92].

To overcome these shortcomings, the combination of AI and QSAR models is being evaluated and trained to learn from relatively sparse, noisy, and complex, high-dimensional data, to better capture patterns in structure–function relationships of PROTACs, leading to better prediction of degrader constructs, with drug-like attributes, against specific target proteins. This new paradigm in AI-driven QSAR has resulted in novel predictive algorithms, including graph neural networks (GNNs), generative models for linkers, multi-task and transfer learning algorithms and explainable AI [93,94], along with the use of transformers to process molecular representations for high-accuracy predictions of PROTAC behavior [95].

GNNs modeling describes molecules as graphs, with nodes representing atoms and edges representing bonds. This modeling approach can accurately capture the topological and relational information within PROTACs and learn representations directly from molecular structure. Recent publications [96] have reported that GNNs can accurately predict ternary complex formation and degradation potential across multiple E3 ligases. More recently, a 3D graph neuronal network, DegradeMaster is introduced, which utilizes 3D spatial information to further improve PROTAC degradation effectiveness [97,98].

Composition and geometry of linker regions in degrader structures are critical to degrader function, affecting ternary complex formation, solubility and cell permeability, and overall degradation efficiency. Novel AI models like DeLinker [99] and DiffLinker [100] employ deep generative techniques to allow for the design of optimal linkers. These models learn linker patterns from large chemical databases and generate novel candidates based on specific spatial constraints, thus reducing the number of experimental iterations. Deep learning models capable of predicting multiple endpoints, such as degradation efficiency, off-target effects, and ADME properties, offer a comprehensive approach to degrader optimization. Multi-task models leverage shared information across tasks to improve learning efficiency, especially within the limitations of available data [101].

Transfer learning approaches are also obtaining traction; these reuse available knowledge from related drug discovery tasks and apply such knowledge to new conditions. For example, models pretrained on large chemical datasets can be fine-tuned on smaller PROTAC-specific datasets, improving performance on degradation tasks. As degraders enter the clinical space, interpretability becomes crucial. Explainable AI techniques like SHAP (SHapley Additive exPlanations) and attention-based mechanisms allow researchers to identify which molecular substructures or linker properties affect degradation efficiency the most, allowing for feedback to guide in the rational design of degrader structures [102,103,104].

## 8. Predicting ADMET and Toxicity Profiles

A major reason for therapeutic drug failure lies in undesirable Absorption, Distribution, Metabolism, Excretion, and Toxicity (ADMET) properties. Predicting these critical drug properties early in the development process allows for significant de-risking in drug development, reducing costs, and accelerating the delivery of significant therapies. Until recently, ADMET properties have been evaluated through laborious and expensive experimental methods, frequently involving in vitro and in vivo studies. These methods can be resource-intensive, time-consuming, and can be applied to a limited number of compounds. Nevertheless, with new developments in Artificial Intelligence (AI), ADMET drug properties can now be predicted in silico, leading to significant reduction in costs and time, and potential de-risking of drug failure [105,106].

AI, through the combination of machine learning (ML) and deep learning (DL), has emerged as a promising tool in overcoming the ADMET bottleneck in drug development. By leveraging vast datasets of chemical structures and their associated experimental ADMET and toxicity data, AI models can learn complex patterns and make highly accurate predictions of ADMET properties for novel compounds [106,107].

Most ML approaches in ADMET prediction are dependent on algorithms such as Random Forest, Support Vector Machines (SVM) and Gradient Boosting Machines (GBM). These models require cautiously curated datasets of molecular descriptors and associated ADMET characteristics. Otherwise, DL algorithms, especially GNNs, have shown great success. GNNs let the processing of molecular structures as graphs, capturing complex relationships between atoms and bonds, which is vital for understanding the process of molecular interaction with biological systems. GNN-based platforms, when trained on widespread datasets, can provide fast and accurate predictions across a wide range of ADMET properties. Additional methods, such as AutoML, automate the predictive process by choosing the best machine learning algorithms and augmenting their hyperparameters, making AI-driven ADMET prediction more accessible and efficient. Furthermore, newly emerging research focuses on the use of quantum machines in ADMET predictions, promising significant improvement in performance and prediction accuracy [108,109,110,111].

A series of AMET prediction software packages, including both open source and commercial ones, is provided in Table 1 and the list of available databases for training various AI models is provided in Table 2.

## 9. Assessing Model Validity and Reliability

Model endorsement is important in QSAR development because it confirms that predictive models are precise and generalizable. There are several internal validation practices such as leave-one-out (LOO), k-fold cross-validation, and bootstrapping [131] are regularly used to evaluate a model’s stability and validity during training. Internal validation alone is not enough so different external authentication using an independent dataset is crucial to validate that the model performs better on unseen compounds. For this, several Metrics such as the coefficient of determination (R2), cross-validated R2 (Q2), root mean square error (RMSE), and concordance correlation coefficient (CCC) are greatly used to evaluate the performance. OECD guidelines require a model to pass both internal and external validation criteria to be considered predictive [3,132].

Another keystone of QSAR model trustworthiness is the Applicability Domain (AD), the chemical space in which a model can make consistent predictions. There exists several techniques for this, including the leverage approach (Williams plot), distance-based methods (Euclidean or Mahalanobis distance), and ensemble-based probability approximations [133]. ADAN (Applicability Domain Analysis) and other tools like Ambit Discovery and QSAR Toolbox deals with practical applications to consider whether query compounds fall within the domain. In addition, Y-randomization (or response permutation testing) is used to identify chance correlations by randomly shuffling the activity data and rebuilding the model several times. It is expected a valid model should perform implicitly better than the randomized complements. Recently, Monte Carlo sampling, double cross-validation, and consensus modeling have further boosted model robustness and reproducibility [134]. These validation pipelines are specifically important for supervisory approval in fields like environmental toxicology and medicines, where consistency and transparency are principal.

While model validation is an established requirement in QSAR, inadequate or superficial validation often leads to overfitting and misleading performance metrics. Internal validation methods such as k-fold cross-validation, bootstrapping, and Y-randomization assess robustness within the training dataset, but they cannot fully guarantee generalizability [135]. External validation using independent test sets is considered the gold standard, as emphasized by Tropsha and Golbraikh [136]. Moreover, the OECD principles for QSAR validation recommend defining the applicability domain (AD), which delineates the chemical space where predictions are reliable [137]. To mitigate failure cases, best practices include combining internal and external validation, applying consensus modeling, and benchmarking against well-curated datasets. Practical adoption of these recommendations can reduce model overestimation and enhance regulatory acceptance.

Despite significant progress, ML and DL approaches face critical limitations. ML methods, though robust against noise, often struggle with interpretability and may overfit in small datasets. DL models, while powerful in capturing nonlinear renonlinears, typically require large, high-quality datasets and are vulnerable to bias from imbalanced training data [138]. Furthermore, most reported QSAR successes underrepresent negative results, limiting awareness of failure cases [139]. Addressing these challenges requires transparent reporting, model explainability, and rigorous external validation.

## 10. Software, Databases and Computational Platforms

With the growth of computational resources and several AI tool, many platforms now play a vital role in the rationalization of computational drug discovery pipelines, especially in QSAR modeling as presented in Table 3. RDKit (2019 release) [28], is a very popular open-source cheminformatics toolkit in Python and is being extensively used for calculating molecular descriptors, generating molecular fingerprints, and performing structure-based filtering. Similarly, PaDEL-Descriptor (2011 release) [27] is another widely used tool for computing more than 1,400 descriptors and fingerprints, making it suitable for both academic and industrial workflows. KNIME (2015 release) [49], is a very popular graphical workflow-based analytics platform that permits users to build and automate modeling pipelines by drag-and-drop components which later integrates the tools for data preprocessing, machine learning, and molecular descriptor generation [140,141].

DeepChem (2018 release) [57] and Chemprop (2019 release) [58] are other Deep Learning and graphical based approach that has become increasingly admired. DeepChem use real executions of deep neural networks, graph convolutional networks (GCNs), and multitask learning frameworks custom-made for molecular property prediction. In contrast, Chemprop (2019 release) built with PyTorch (release 2017), is adjusted for message passing neural networks and is predominantly operative for structure-activity relationship tasks using SMILES strings [30]. There are several public databases like ChEMBL2017 release) [142], ZINC (2012 release) [143], and PubChem (2004/2006 release) [144] that provide broad curated chemical and bioactivity data, empowering large-scale QSAR and machine learning studies [145]. These are complemented by different cloud-based resources like Google Colab (2018 release) [57] and AWS SageMaker (2017 release) [146] that makes it possible to run QSAR workflows at scale without demanding local hardware. On the other hand, cheminformatics incorporation with cloud-based notebooks accelerates reproducible investigation and easy distribution of computational pipelines, profoundly decreasing the barrier to entry for scholars all over the world [147]. 

## 11. Challenges, Ethical Considerations and Regulatory Aspects

AI-driven drug discovery and development represent a promising platform for accelerating the development of new medicines; at the same time, such technology brings significant challenges, ethical considerations, and evolving regulatory aspects that must be carefully navigated [148,149]. Among the many challenges, key issues are data quality and availability. AI models require extensive data for model training; furthermore, these data must be of high quality. In the drug discovery space, the availability of large amounts of quality, that is comprehensive and unbiased data from various sources, is a major hurdle. Frequently the available datasets can be incomplete, noisy or proprietary, making it difficult to integrate and use effectively for properly training AI-models. AI models trained on narrowly defined datasets will likely not have much utility as minor variations in training parameters or input data can lead to different outputs, raising concerns about reproducibility and reliability. Additionally, certain data, especially for pharmacokinetic and toxicity analysis, may be biased toward particular chemical scaffolds or patient demographics, limiting model generalization.

Modeling complex biological systems to determine how a drug will interact with such systems presents major challenges; indeed, modeling even simpler systems, such as cellular structures, can be overwhelming given the complex interrelations between the cellular components and the dynamic nature of these systems. Commercially, the employment of AI in drug discovery requires considerable investment in advanced computational hardware and software, infrastructure, and skilled workforce with proficiency in both AI/machine learning and pharmaceutical sciences. Such investments can be a barrier for smaller companies or research institutions. Furthermore, the integration of AI technology into existing drug discovery and development pipelines and workflows within pharmaceutical organizations can present significant integration challenges and, potentially, low adoption.

From ethical considerations, AI models trained on biased data can perpetuate and even reinforce existing disparities in healthcare. If datasets are not representative of diverse patient populations, the AI’s predictions may lead to inequitable outcomes in drug efficacy and safety for underrepresented groups. The use of patient-level data, including genomic, electronic health record data, and clinical trial data for AI training must be governed by strict privacy protections and ethical standards of informed consent to prevent re-identification of anonymized data, data breaches, or unauthorized secondary use of the data.

The nature of some AI models raises ethical concerns regarding accountability. If an AI-driven drug leads to adverse outcomes, it can be challenging to pinpoint the exact reasons for the AI’s decision, making it difficult to allocate responsibility. Likewise, when AI systems are used to lead therapeutic decisions or impact clinical trial designs, questions of accountability arise; therefore, inventors must ensure traceability of model inputs, outputs, and decision logic. The regulatory landscape as it applies to the use of AI in drug development is as challenging as the ethical issues discussed above. Meanwhile, regulatory bodies worldwide, such as the FDA (US) and EMA (Europe), are working to develop appropriate guidelines for AI-driven drug development [150]. This is a rapidly evolving and changing area, and companies face the challenge of complying with frameworks that are still conceptual in some respects and, at best, partially defined.

Regulatory agencies are increasingly incorporating AI and QSAR into safety assessment. While still in the very early stages, both FDA and EMA currently accept QSAR assessments under ICH M7(R2) for the identification and control of DNA-reactive impurities [151]. Additionally, the FDA’s Center for Drug Evaluation and Research (CDER) has partnered with the National Center for Toxicological Research (NCTR) through the ‘SafetAI Initiative’ to develop and validate AI-based QSAR models for predicting key safety endpoints, including hepatotoxicity, carcinogenicity, and cardiotoxicity [152]. This effort not only strengthens internal expertise but also establishes standards against which industry submissions can be evaluated.

Interpretability remains a regulatory priority. Approaches such as SHAP (Shapley Additive Explanations) have been successfully applied to QSAR toxicity models, where they highlight the molecular substructures most responsible for predictions, thereby improving transparency and confidence in model use [153,154]. These developments indicate a convergence between regulatory acceptance and explainable AI, supporting safer integration of AI-enhanced QSAR into decision-making.

When utilizing AI Models to support regulatory submissions, regulatory agencies invariably require robust validation and verification of AI models that facilitate regulatory decision-making, especially in relation to drug safety, effectiveness, and quality. Regulatory requirements will necessitate demonstration of data integrity and traceability, and verifiable model performance. Additionally, regulatory submission, relying on any AI-driven decisions, will require that such AI models be extensively validated using external datasets and benchmarked against established clinical standards and must contain documentation details on data source, preprocessing, model architecture, and performance metrics. Regulatory agencies are likely to shift towards a risk-based approach, where the level of scrutiny for an AI model depends on its “context of use” (COU) and the potential risk associated with its output. Eventually, data used to train AI models must be “fit for use,” meaning that the data must be applicable, reliable, and of sufficient quality to support the intended regulatory purpose. Furthermore, regulators are going to demand a greater need for transparency and explainability in AI models, especially those impacting on critical regulatory decisions.

For continuous learning AI models that adapt over time (“self-evolving”), regulatory agencies will be faced with ensuring ongoing model validation and monitoring to prevent “model drift,” such that the model’s performance does not degrade or become unreliable. For situations in which AI is being used in pharmacovigilance to identify adverse drug events and safety signals from large datasets. Regulators should make sure that the AI model used in this crucial post-marketing phase is precise and real. Correspondingly, if AI is applied in patient stratification, simulation of trial outcomes, and identification of relevant biomarkers, it is important that such applications do not introduce bias or compromise statistical integrity.

## 12. Conclusions

The future direction of AI in drug development will be characterized by several key trends and advancements. Firstly, AI will continue to revolutionize the early stages of drug development through the use and analysis of vast datasets, including genomics, proteomics and clinical studies to efficiently identify and validate promising drug targets. This process will allow drug developers to focus and manage resources on the most relevant proteins and molecules involved in disease pathways. Additionally, generative AI and deep learning models, including variational autoencoders, generative adversarial networks and large language models, will become even more sophisticated, especially with appropriate training, in the design of novel compounds with drug-like properties, predicting molecular interactions, and optimizing chemical structures to improve efficacy, simulating biological responses and reducing off-target interactions and side effects, thus minimizing toxicity.

AI will also be deployed for the rapid identification of existing drugs that can be repurposed for new indications; therefore, shortening the development timeline relative to the discovery and development of entirely new compounds and also proposing potential synergistic drug combinations.

AI will play a more dominant role in clinical trial design by analyzing historical data, resulting in ideal trial protocols, optimal endpoints, and timelines [155]. It will be used to simulate trial scenarios and allow for fine-tuning of dosage and treatment duration. AI algorithms will be used to analyze available medical data to determine suitable patient candidates for clinical trials and ensure more diverse and representative cohorts. The use of AI in trial design and management will enable real-time modifications to trials, allowing adjustments to dosages or patient cohorts based on interim results, increasing efficiency and supporting decentralized clinical trials. Furthermore, AI algorithms will enable better patient stratification via the analysis of genomic, proteomic, and clinical data, allowing for targeted treatments and personalized medicine approaches and even predicting individual patient responses to specific therapies for customized treatment plans for optimal outcomes.

To conclude, we presented the use of QSAR modeling with artificial intelligence, molecular docking, and molecular dynamics simulations that have meaningfully upgraded the field of computational drug discovery. Classical regression methods, which remain foundational, are now being conveyed by robust machine learning and deep learning approaches proficient in seizing complex, nonlinear hips in chemical data. While rigorous validation practices continue to ensure their scientific and regulatory credibility, the implementation of cloud-based platforms and open-source tools has made these technologies more accessible. As the field progresses, the conjunction of data-driven methods with mechanistic modeling may be the key to building more robust, reliable, and ethical drug discovery pipelines.

## Figures and Tables

**Table 1:** List of ADMET prediction software programs.

Name	Type	Key Features
ADMETlab (v3.0)	Open-Source	On-line multi-endpoint ADMET & toxicity prediction [112].
pkCSM (2015 release)	Open-Source	Graph-based for ADMET classification [113].
SwissADME (2017 release)	Open-Source	On-line tool for ADME, physicochemical, and drug-likeness [114].
ProTox-II (v2, 2018)	Open-Source	Toxicity endpoints including LD50, hepatotoxicity [115].
T.E.S.T. (v5.1.1)	Open-Source	EPA tool for QSAR-based toxicity estimates [116].
DeepChem (v2.x)	Open-Source	Python ML/AI library for molecular modeling [2].
VEGA QSAR (v1.2.3)	Open-Source	Rule-based QSAR toxicity predictor [117].
AdmetSAR (v2.0, 2019)	Open-Source	Predictive model for ADMET endpoints [118].
ADMET-AI (2023 release)	Open-Source	ML-based tool for fast and accurate ADMET predictions [106].
CtoxPred3 (v3)	Open-Source	In silico prediction of peptide toxicity
Schrödinger QikProp (v6.2)	Commercial	50+ ADME properties, integrated in Schrödinger [119].
ACD/Percepta (v2023.1)	Commercial	Physicochemical, ADME, and toxicity predictions [120].
DEREK Nexus (v6.x)	Commercial	Rule-based toxicology and safety predictions [121].
TOPKAT (BIOVIA) (v6.2)	Commercial	QSAR-based toxicity (mutagenicity, carcinogenicity) [122].
ADMET Predictor (v11.5)	Commercial	175+ ADMET properties and metabolism simulation [123].
StarDrop (v7.3)	Commercial	ADMET modeling with compound prioritization [124].
ChemAxon’s cxcalc (v23.15)	Commercial	Command-line ADME prediction tool [125].
ToxTree (v2.6.13)	Commercial	Decision tree-based toxicity analysis [126].

**Table 2:** ADMET databases for AI model training.

Tox21/ToxCast	Large public databases with toxicity screening data for thousands of compounds [127].
PubChem BioAssay	ADMET-related assay data and curated datasets for ML model development [128].
ChEMBL	Contains bioactivity data including absorption, CYP inhibition, and off-target toxicity [129].
DrugBank	Pharmacokinetic and toxicity data for approved drugs, useful for validation and model tuning [130].

**Table 3:** Computational Tools and Their Applications in QSAR-Based Drug Discovery.

Tool/Database	Application	Reference
RDKit (2019 release)	Molecular descriptor generation, structure processing	[28]
KNIME (2015 release)	Workflow automation and model building	[49]
ChEMBL (2017 release)	Bioactivity database for QSAR modeling	[129,142]
DeepChem (2018 release)	Deep learning platform for cheminformatics	[57]
ZINC Database (2012 release)	Commercially available compound repository	[143]
Chemprop (2019 release)	SMILES-based deep learning QSAR modeling	[58]
Google Colab (2018)	Cloud-based Python notebook environment	[57]

## Data Availability

No new data were created or analyzed in this study. Data sharing is not applicable to this article.

## Abbreviations

QSAR, Quantitative Structure–Activity Relationship; QSPR, Quantitative Structure–Property Relationship; AI, Artificial Intelligence; ML, Machine Learning; DL, Deep Learning; MD, Molecular Dynamics; CNN, Convolutional Neural Network; RNN, Recurrent Neural Network; GNN, Graph Neural Network; GCN, Graph Convolutional Network; SVM, Support Vector Machine; RF, Random Forest; kNN, k-Nearest Neighbors; PLS, Partial Least Squares; MLR, Multiple Linear Regression; PCR, Principal Component Regression; PCA, Principal Component Analysis; RFE, Recursive Feature Elimination; RMSE, Root Mean Square Error; RMSD, Root Mean Square Deviation; LOO, Leave-One-Out; AD, Applicability Domain; ADAN, Applicability Domain Analysis; SHAP, SHapley Additive exPlanations; LIME, Local Interpretable Model-agnostic Explanations; HOMO, Highest Occupied Molecular Orbital; LUMO, Lowest Unoccupied Molecular Orbital; SMILES, Simplified Molecular Input Line Entry System; GANs, Generative Adversarial Networks; VAEs, Variational Autoencoders; PROTAC, PROteolysis Targeting Chimera; ADMET, Absorption, Distribution, Metabolism, Excretion, and Toxicity; GBSA, Generalized Born Surface Area; MM/PBSA, Molecular Mechanics/Poisson–Boltzmann Surface Area; OECD, Organisation for Economic Co-operation and Development; FDA, Food and Drug Administration; EMA, European Medicines Agency; REACH, Registration, Evaluation, Authorisation and Restriction of Chemicals; KNIME, Konstanz Information Miner; CHARMM, Chemistry at HARvard Macromolecular Mechanics; GROMACS, GROningen MAchine for Chemical Simulations; NAMD, Nanoscale Molecular Dynamics.
